# Measles Vaccine Boosters in Immunocompromised Adults to Reduce Morbidity and Mortality From Acute Measles Infection: A Scoping Review

**DOI:** 10.7759/cureus.115440

**Published:** 2026-08-30

**Authors:** Isaac Price, Perry K Smith, Abigail Tan, Annieliesa Suarez-Rocha, Mariya Baig, Zaida Athouriste, Carole Aziz, Nahinoon Razzaque, Sarah Sadik, Sebastian Breeland, Beth Gilbert, Joseph S De Gaetano

**Affiliations:** 1 Family Medicine, Dr. Kiran C. Patel College of Osteopathic Medicine, Nova Southeastern University, Fort Lauderdale, USA; 2 Osteopathic Medicine, Dr. Kiran C. Patel College of Osteopathic Medicine, Nova Southeastern University, Fort Lauderdale, USA

**Keywords:** booster, cancer, diabetes, hiv, ibs, lupus, measles, vaccine

## Abstract

Measles is a highly contagious viral illness associated with significant global mortality, despite the availability of an effective vaccine. As vaccine hesitancy continues to increase following the COVID-19 pandemic, combined with a lack of measles-specific antiviral agents, the risk of outbreaks and epidemics remains elevated. Immunocompromised individuals have a higher risk of serious complications from measles, especially because the live-attenuated measles, mumps, and rubella vaccine may be contraindicated in severely immunocompromised patients. As such, these individuals depend on herd immunity. This scoping review aims to identify the potential need for a measles booster dose among immunocompromised adults in the United States by providing an overview of current vaccine indications and outcomes. EMBASE, Web of Science, and Ovid MEDLINE were searched using search terms specific to measles booster doses and immunocompromised adults. In total, 2,335 articles were imported to Rayyan, and after a two-tiered review, seven articles met the full inclusion criteria. The results revealed that individuals with chronic immunocompromising illnesses were often not fully protected against measles, leaving them vulnerable to infection. The findings from this review suggest that clinicians may consider a booster dose of vaccine for immunocompromised individuals to help restore their protection against measles. Meanwhile, further research utilizing larger sample sizes and diverse patient populations is warranted based on these findings. We believe that additional studies should aim to clarify current guidelines to improve the care of immunocompromised patients who may be exposed to measles.

## Introduction and background

Measles cases, despite the availability of an effective vaccine, spiked worldwide during 2017, resulting in the death of 110,000 individuals. Increased mortality was seen among both children and immunocompromised individuals [1]. Control efforts in the United States (U.S.) have been relatively ineffective at reducing measles cases, despite the declaration of measles elimination in 2000. Cases of measles in the U.S. continued to rise during 2024 [2,3]. Combined with the virus’s ability to be both immunosuppressive and erase the immune system’s memory of other pathogens, measles has the potential to negatively impact both immunocompromised and immunocompetent individuals [4]. Before vaccinations were available in 1963, the U.S. experienced four million cases and 450 deaths, as compared with current estimates of 20 million new cases and 100,000 deaths worldwide annually [2]. With the rapid introduction of the measles vaccine in the U.S. and the gradual introduction in Great Britain and other European countries, the prevalence of the disease declined from a common disease to a rarity [5]. Without a vaccine, measles epidemics would be expected to occur every two to five years, costing the U.S. $3.6 billion annually [1]. The potential for epidemics amid increased patient vaccine refusal and the lack of specific antiviral treatments highlight the importance of robust individual immunity to the measles virus [1,4,6,7].

To eradicate measles in the U.S., mass vaccination is instrumental in lowering disease burden [8]. Long-lasting vaccine-induced immunity appears to be the best possible defense against measles, and severe immunosuppression is a contraindication for the live attenuated measles, mumps, and rubella (MMR) vaccine and the measles, mumps, rubella, and varicella (MMRV) vaccine. As such, these populations depend on herd immunity for protection [9]. In those with previously compromised immune systems, such as children and adults who have been cured of cancer, a more rapid decline in measles antibodies has been found, indicating a potential need for repeat vaccinations beyond the initial two-dose regimen [9]. This scoping review aimed to evaluate the efficacy of a measles booster dose among immunocompromised individuals aged 21 years and older who were eligible for vaccination in the U.S.

Understanding the pathophysiology of measles infection is important for understanding the need to prevent the disease through vaccination. The measles virus is a negative-sense, single-stranded ribonucleic acid virus that is transmitted via the respiratory route [3,10]. It is highly infectious, with 94% of the population requiring vaccination to achieve herd immunity [11]. Most patients infected with the virus are children, with a median age of nine years. Additionally, over 90% of patients infected with measles are unvaccinated [12]. Measles enters the body through the respiratory tract [3,13]. The measles virus targets respiratory tract dendritic cells and migrates to the lymphatic system, where the infection spreads throughout the body [1]. The incubation period is approximately 10 days. Fever and rash start to appear after approximately 14 days [1]. Cough and Koplik spots are characteristic features of measles infection [1]. Modified measles is a notably milder and less contagious variant of the disease that can occur among patients who have been vaccinated or previously infected [8].

Measles vaccinations have drastically reduced the number of annual cases [2]. Cases and deaths within the U.S. have declined from an annual count of 535,600 to 142,300 between 2001 and 2018, respectively [8]. Severe complications, including hyponatremia, pneumonia, and death, are notably higher among hospitalized patients [14]. Supportive care is the prevailing approach to managing measles, as there is no specific U.S. Food and Drug Administration-approved treatment [15]. As such, vaccination has been vital to eradicating this disease [10].

Measles is currently on the rise again in the U.S. [2,16]. Despite the existence of an effective vaccine, low vaccination rates in the U.S. are contributing to this increase [12]. This is despite evidence that the adverse effects of the MMRV are rare [17]. This trend is especially troublesome for immunocompromised patients, as they can progress to develop life-threatening complications, including but not limited to pneumonitis and encephalitis [18]. While most current U.S. literature on measles focuses on risks in the pediatric population, individuals aged 21 years and older who are immunocompromised in the U.S. face increased risk of contracting the virus as well. Immunocompromised individuals experience a more rapid decline in measles antibody levels after vaccination than immunocompetent individuals [9]. Increased secondary MMRV failure has been attributed in a significant number of U.S. cases to waning vaccine immunity, which could further increase the risk of measles infection among immunocompromised individuals [19].

Measles infection in immunocompromised adults is a serious concern due to the lack of antiviral treatments and the increased risk of severe complications [15]. Because a 95% herd immunity threshold is needed to protect a population from measles, the measles booster could benefit not only immunocompromised individuals but also the population at large [20]. Given the current literature on measles and the annual increase in cases, there is a lack of information on the efficacy of a measles booster dose among immunocompromised individuals aged 21 years and older in the U.S. Given the increased risk of immunocompromised adults contracting measles due to declining herd immunity in the U.S., we conducted a scoping review to assess the potential need for immunization protocols to protect this vulnerable population.

## Review

Inclusion and exclusion criteria

For our inclusion criteria, we included articles published between 2015 and 2025. U.S.-only publications were included; English-language-only articles were included. Cohort, case-control, and randomized controlled trials were included. Studies with adults aged 21 years and older were included, and the study population must consist of patients with an immunocompromising disease in the experimental group. Each study population was limited to a single immunocompromising disease being evaluated (i.e., one study cannot include both irritable bowel syndrome and diabetes, but it can include either one or the other). These criteria were intended to narrow the number of studies to allow a close examination of the most relevant studies for potential directions of future research on vaccines for adults in the U.S.

For our exclusion criteria, case reports, expert opinions, or other opinion pieces were excluded. Additionally, scoping and systematic reviews were excluded, as were conference abstracts for which the full text could not be obtained. When going through the searches in Rayyan (Cambridge, MA, USA), we included exclusion labels, such as a + (positive) label for an exclusion criterion or a − (negative) label for an inclusion criterion, as the reason we did not include the article. All search results were imported into Rayyan using compatible formats. Duplicate records were removed prior to screening. Study selection was conducted in two tiers, followed by further critical appraisal of studies selected for inclusion in Tier-II.

First, for Tier-I (title and abstract screening), review authors #1 and #2 screened titles and abstracts against the inclusion and exclusion criteria. Each record was marked as include, exclude, or maybe. Custom exclusion labels, in accordance with our inclusion and exclusion criteria (e.g., irrelevant population, incorrect study design), were applied to ensure consistency. Disagreements were flagged by Rayyan and resolved by a tiebreaker from review author #3, with brief reasons for exclusion provided for the abstracts we excluded. Second, for Tier-II (full-text screening), articles marked as "include" or "maybe" in Tier I underwent a more in-depth review by authors #1 and #2 to confirm their eligibility for data synthesis. Reviewers marked each text for exclusion or inclusion based on our criteria (outlined above). Any disagreements were resolved by author #3. The final set of included studies for synthesis in our review paper was exported from Rayyan to Excel (Microsoft Corp., Redmond, WA, USA) to create a data summary table. The Tier II articles also underwent critical appraisal using Joanna Briggs Institute (JBI) appraisal checklists. Then, a Preferred Reporting Items for Systematic Reviews and Meta-Analyses extension for Scoping Reviews (PRISMA-ScR) flow diagram was used to document the number of records identified, screened, excluded, and included in the final synthesis in an easy-to-digest format for inclusion in this scoping review [21].

Data extraction and synthesis

Data extraction was conducted to organize data for a structured summary table in Excel after study selection was completed in Rayyan, in accordance with the inclusion and exclusion criteria for our population. The table divided parts of the articles into easily comparable fragments. The extracted variables included the author and year, the study's aim, the study population, the study methods, and the results and conclusions. We used this organization to identify patterns and trends in the articles we included and to synthesize the ideas found in those papers into an understandable narrative. The final extraction table is presented alongside the paper's review and synthesis to demonstrate compliance with JBI standards. The synthesis of our review aimed to identify trends, knowledge gaps, and questions for future research.

Critical appraisal

A critical appraisal of the studies gathered was conducted following the Tier II review to ensure our review was based on studies that contained reliable evidence. The JBI Critical Appraisal tools were used to systematically classify the final articles as high, moderate, or low quality [22]. A different checklist was used for each type of study design. Six reviewers assessed study design quality, bias/validity, methodological clarity, and transparency using the JBI checklists. Consensus on each article was reached by performing the JBI critical appraisal with multiple reviewers and comparing results.

The JBI appraisal process was used to identify low-quality studies and to indicate whether a study was high, moderate, or low quality, based on responses from multiple collaborators, to determine whether it was of sufficient quality to be included in our final data synthesis.

To accurately represent our data, we had to ensure that our articles were high-quality and scientifically sound. Papers with low validity could alter our data analysis and lead to misrepresentation. Studies, including those we selected for our scoping review, underwent a critical appraisal to assess the extent to which they had considered potential bias in their design, conduct, or analysis. Critical appraisal tools facilitate this evaluation. We used the JBI critical appraisal tool checklists to evaluate the articles identified as relevant to our study in our Tier-II review. We selected seven articles through our Tier-II review, and each article was assigned to one author for in-depth review using the appropriate JBI checklist based on the study's methodology. The studies assessed were categorized into three risk levels based on the percentage of questions answered positively in the JBI checklist. Scores below 50% indicated high risk, scores between 50% and 70% indicated moderate risk, and scores above 70% indicated low risk. All seven articles assessed scored above 70% in the JBI criteria and were included in our review. The relevance and quality of each study were thoroughly discussed to select the final articles for the review, and all seven were included.

Methods

This scoping review was conducted using the JBI methodology and reported in accordance with PRISMA-ScR guidelines. This scoping review examined adults aged 21 years or older with immunocompromising diseases, including but not limited to cancers, human immunodeficiency virus (HIV), transplantation, inflammatory bowel disease (IBD), Crohn’s disease, rheumatologic diseases, and diabetes. Only populations within the U.S. were examined.

The inclusion criteria for this review, outlined above, included U.S.-only publications in English, published between 2015 and 2025. Other criteria included research studies using cohort, case-control, and randomized controlled designs. Manuscripts excluded from this analysis were case reports, expert opinions or other opinion pieces, scoping reviews, and systematic reviews. We also did not include conference abstracts for which we could not find the full text.

The objective of this scoping review was to assess whether there is a need for a measles booster vaccine among immunocompromised adults in the U.S. This review analyzed current indications and outcomes for measles vaccination and booster dosing in this population.

Eligibility criteria

Research studies were included in the review if they involved immunocompromised adults aged 21 years or older in the U.S. and reported measles immunity or seroprevalence or demonstrated an immune response to measles vaccines. Studies that were published between 2015 and 2025 were considered for review. To narrow our study set to the most relevant articles, we established inclusion and exclusion criteria (as outlined above).

Search strategy

Our scoping review systematically searched the literature using Ovid MEDLINE, EMBASE, and Web of Science with Boolean operators (AND, OR, *) and search terms related to measles booster doses and immunocompromised adults. Terms such as “booster dose”, “measles mumps rubella”, “immunocompromised”, and "autoimmune" were also searched. The tables below provide an overview of the results by search term for each database queried (see Appendices).

Authors #6-#10 conducted a literature search on the epidemiology/prevalence, pathophysiology, morbidity and mortality, treatment, and prevention of measles. Each author was responsible for locating four unique articles based on their search topic. The databases used are listed in our appendix below: Ovid MEDLINE, Web of Science, and EMBASE. The search findings were inserted into EndNote and screened for duplicates. One duplicate was identified, so author #7 located an additional article to use for the background section.

A total of 2,335 references were imported to Rayyan. Duplicate articles were removed before the screening. Authors #1 and #2 independently conducted a blind Tier I review of the titles and abstracts of 1,226 articles. After authors #1 and #2's decisions, the blind review was lifted, and author #3 served as a tiebreaker for any disputed articles. After the Tier I screen, 1,205 articles were excluded for failing to meet the inclusion criteria. Twenty-two articles were initially approved. One article was excluded after confirming it did not meet the initial inclusion criteria of being written in English. Authors #4-#10, excluding author #5, collected the full text for 20 articles. Author #5 uploaded the full-text articles onto Rayyan to begin the Tier II review. Authors #1 and #2 independently conducted a blind Tier II review of the full-text articles. The blind review was lifted after authors #1 and #2 completed their reviews, and author #3 had the decisive vote for any contested articles. This final review resulted in the exclusion of 15 articles for failing to meet the screening criteria. Seven articles were approved and included in this review. The article selection process was documented using a PRISMA-ScR flow diagram (Figure 1). A summary of the characteristics of the included studies is included in Table 1.

**Table 1:** Characteristics and findings of the included studies HIV: human immunodeficiency virus, MMR: measles, mumps, and rubella, ELISA: enzyme-linked immunosorbent assay, SD: standard deviation, SE: standard errors, HCT: hematopoietic cell transplant, IBD: inflammatory bowel disease, PCP: primary care provider, EMR: electronic medical records, TNF: tumor necrosis factor, U.S.: United States, RCTs: randomized controlled trials

Author, year	Aim of study	Study setting	Sample description	Methods (study type/design)	Main findings	Limitations	Study conclusions
Rearigh et al., 2020 [23]	Establish the current immunity levels to measles among HIV- positive individuals in the U.S. and identify risk factors linked to measles seronegativity	Academic HIV clinic in Omaha, Nebraska	351 participants with ages ranging from 20-74 years. Average age was 48 years, and 12% were born before 1957. Among the participants, 77% were male, 53% were Caucasian, and 81% were born in the U.S.	Cross-sectional study. Participants had to complete a questionnaire indicating their demographics, provide their medical records, and undergo screening for measles IgG antibody. To identify risk factors for measles seronegativity, researchers used simple and multivariable logistic regressions	Among individuals with HIV, only 70% carry measles immunity, which is less than the 95% needed to obtain herd immunity. The younger population and those born after 1957 were the most likely to be negative for measles IgG. Seropositivity was associated with previous measles infection	The study is limited in generalizability due to its conduct at a single HIV clinic. Self-reported vaccinations and clinical records may not be accurate. The study does not follow participants to keep track of immunity over time and only measures measles immunity at one point in time	Measles immunity was found to be much lower than needed to achieve herd immunity. Individuals born after 1957 were more likely to be seronegative for measles, with no other notable risk factors. Based on that, it was concluded that HIV- positive individuals who are at risk of exposure to measles should be considered for expanded screening for measles immunity
Andrews et al., 2025 [24]	To determine the proportion of adults with HIV in a large U.S. clinic who had measles immunity and identify factors associated with lack of immunity	Parkland Health HIV Clinic, Dallas, Texas, USA	13,622 adults (≥18 years) with HIV seen from 2015-2025	Retrospective cohort study using EHRs	11.2% of patients had measles immunity; factors like younger age, white race, and low CD4 count (<200 cells/µL) were linked to not being immune to measles	Only one clinic studied. Only past medical records were used. Only one MMR vaccine dose was documented rather than the full series	Most HIV patients were not immune to measles; clinics should check immunity and vaccinate patients that are not immune
Marquis et al., 2021 [25]	Identify a point prevalence estimate of protective measles and mumps antibodies among ambulatory patients with cancer	Seattle Cancer Care Alliance/Fred Hutchinson Cancer Research Center in Seattle, Washington	959 adult patients with cancer; 510 (53%) were males, and the mean (SD) age at sample collection was 65 (15) years. Most patients (576 (60%)) had a malignant solid tumor; 383 patients (40%) had a hematologic neoplasm; 145 (15%) had an HCT history	Analytical cross-sectional study; residual clinical plasma samples were collected from consecutive cancer outpatients over 5 days in August 2019. A commercial ELISA kit was used to measure measles and mumps IgG antibodies. Multivariable Poisson regression with SEs was used to compare subgroups and estimate prevalence ratios while adjusting for confounding variables	Overall measles antibodies were 0.75, and the seroprevalence of mumps antibodies was 0.62. Lowest seroprevalences were among patients with hematologic neoplasms, those with prior history of HCT, and adults aged 30-59 years	IgG seropositivity may be adequate to measure actual immunity, especially for mumps; vaccination history was unavailable; study was limited to patients during a specific period who underwent clinical blood draws, small pediatric population	25% of ambulatory patients with cancer lacked protective antibodies against measles, and 38% lacked protective antibodies against mumps. Deficits in protective antibodies show patients' increased risk during outbreaks and emphasize the need for community- based efforts to increase herd immunity to protect this population
Lin et al., 2022 [26]	To evaluate the immunogenicity of the MMR vaccine in adults post-HCT to determine the ideal number of doses for the MMR vaccine post-transplant and assess the purpose of measuring postvaccination titers	Single-center retrospective study; Sylvester Comprehensive Cancer Center, Miami, Florida, USA	187 patients who received allogeneic or autologous HCT between February 2008 and September 2021, and one dose of the MMR vaccine after their transplant. The three main diagnoses of patients were leukemia, multiple myeloma, and lymphoma	A retrospective study was conducted among adult patients who received allogeneic or autologous HCT between February 2008 and September 2021 and subsequently received at least one dose of the MMR vaccine posttransplant. 409 patients received an MMR vaccine during the period, but 222 were excluded because they lacked serologic data to compare antibody titers before and after vaccination. MMR titers were collected at 14 or more months after HCT. Nonimmune patients, those with negative or equivocal titers, received their first MMR vaccine when they were more than 1 year off immunosuppression and more than 2 years posttransplant. MMR titers were repeated in the nonimmune patients 2 months after their MMR vaccine	The majority of HCT patients achieved protective titers after one dose of MMR. Autologous patients were significantly more seropositive than allogeneic patients for measles and mumps but not rubella. Among patients who did not respond to the first MMR vaccine, those who received a second MMR vaccine seroconverted. There were no observed differences in various factors like demographics and time between transplant and first MMR dose for responders and non-responders	Limitations of the study included having to exclude many patients due to missing serological MMR titer data and not evaluating clinical efficacy due to entailing more follow-up and exposure to an outbreak	Almost all allogeneic HCT patients lacked humoral immunity against MMR, but this immunity could be re-established with MMR vaccination. HCT programs should think about obtaining MMR antibody titers post- vaccination in patients since a second MMR vaccine dose appears to be beneficial in non-responders
Cleveland et al., 2016 [27]	To determine the level of immunity to measles and pertussis in adults with IBD. To recognize factors that may be associated with non-immunity, especially for patients on immune-suppressive therapy	University of Chicago Medicine, a tertiary IBD center in Chicago, Illinois, USA	The sample included 122 adults with confirmed IBD who were 19 to 76 years old, with the median age being 39. Of the 122, 77 had Crohn’s disease, and 45 had ulcerative colitis. The majority of the sample were on a certain immunosuppressive therapy, including anti- TNF, thiopurine, methotrexate, or anti-integrin therapy	The study was an analytical cross-sectional study. Titers from patients with measles and/or pertussis were routinely obtained from February to May 2015. Standard laboratory values were used to define immunity. Specific patient data were collected, including vaccination history, disease duration, therapies used, and age. The study implemented univariate analysis, which included chi-squares, Fisher’s exact test, and odds ratios	Regarding immunity, 83.6% were immune to measles, while 60% of the sample were NOT immune to pertussis. 13.1% were NOT immune to measles, of whom 75% were on immunosuppressive therapy. Data showed that older age (≥50) and longer measles duration (≥10 years) were associated with lower measles titers. Younger patients and those with shorter disease duration had higher non-immunity to pertussis. Vaccination history recall was not reliable in predicting immune status	Since the study was conducted at a single tertiary-care IBD center, it is difficult to assess its generalizability. There was also no multivariate analysis used to control for any confounding factors. Patients self-reported their vaccination history, introducing recall bias. It is also difficult to establish causation as the study was observational in design	The majority of the sample lacked measurable immunity to measles and pertussis. Many of the patients who were not immune to measles were on immunosuppressants, so they could not receive a live booster vaccine. According to the authors, they recommend regularly assessing for immunity to measles and pertussis in IBD patients before receiving immunosuppressive therapy
Desjardins et al., 2021 [28]	The primary objective of this study was to assess the safety of the MMR vaccine in immunocompromised populations when administered within 2 years of a HCT	Individuals who underwent a HCT at Brigham and Women’s Hospital	There were 129 patients in total, with a median age of 61 years, of whom 57% were male; 58% received autologous HCT and were all vaccinated within 2 years. The prevalent hematologic disorders were multiple myeloma, non-Hodgkin lymphoma, and Hodgkin disease. 65% of participants were also on immune-suppressive therapy at the time of vaccination	A retrospective review was conducted in all patients who received an autologous or allogeneic HCT at BWH/DFCI between 1/2017 and 12/2018 and subsequently received the MMR vaccine <2 years after transplant. A separate cohort of patients with multiple myeloma who received autologous HCT between 3/2009 and 1/2014 and were also found to be vaccinated <2 years after transplant while on therapy with thalidomide analog and/or proteasome inhibitor was also included	Ten adverse reactions in 7 patients (5%) were identified within 42 days of vaccination: 6 respiratory tract infections (3 with fever) and 1 rash. The rash was seen in a 37-year-old female who had an allogeneic HCT 542 days before vaccination. She presented with a centrifugal maculopapular rash, confirmed to be caused by the vaccine strain rubella virus. She fully recovered. No other vaccine-associated illness was identified in the cohort after a median follow-up of 676 days	Since the study was retrospective, the authors believed mild adverse reactions may not have been reported in charts and could have been missed. Also, the decision to vaccinate these individuals was made by their physicians, so it is possible that physicians selected relatively healthy patients, potentially biasing the findings. There was no further data collection on immunogenicity since physicians did not consistently order pre- and post-vaccination serologies	MMR vaccine administered ≥300 days post-transplant was well tolerated in carefully selected individuals. In this study, there were no severe outcomes or deaths. Of note, there was one mild case of rubella reported after vaccination
Ramirez et al., 2024 [29]	To determine short-term safety of MMR vaccination in immunocompromised adults with IBD	University of Wisconsin-Madison gastroenterology and PCP, 2011-2023	35 immunocompromised patients with IBD seen between 2011-2023	Retrospective cohort using EMR and Wisconsin Immunization Registry	No short-term adverse reactions despite vaccine contraindications	Small sample size, confounding variables noted but not controlled for	In adults with IBD on immunosuppressive therapy, inadvertent live vaccine inoculation was not associated with clinical/disseminated infection during patient follow-up. More studies are needed to evaluate safety before vaccine guidelines can be changed

A thorough evaluation of the eligible articles identified in the Tier II review was conducted using the JBI critical appraisal tools. Six reviewers were each assigned one article to carry out an independent detailed appraisal of the selected articles. Each article was assessed in depth for research biases, measures of exposure and outcomes, identification and management of confounding variables, validity of results, and overall strength of evidence using the appropriate JBI checklist based on study methodology. Each item was rated as “yes”, “no”, “unclear”, or “not applicable” in accordance with JBI guidelines. Study quality was classified as high, moderate, or low based on the proportion of criteria met.

Data synthesis and results

Data were extracted and organized into a summary table to facilitate comparison among the included studies. Key aspects of each study were categorized to identify major trends, commonalities, and distinctions. Results of these selected articles were documented in adherence to the PRISMA-ScR guidelines and are reported in Figure 1.

**Figure 1:**
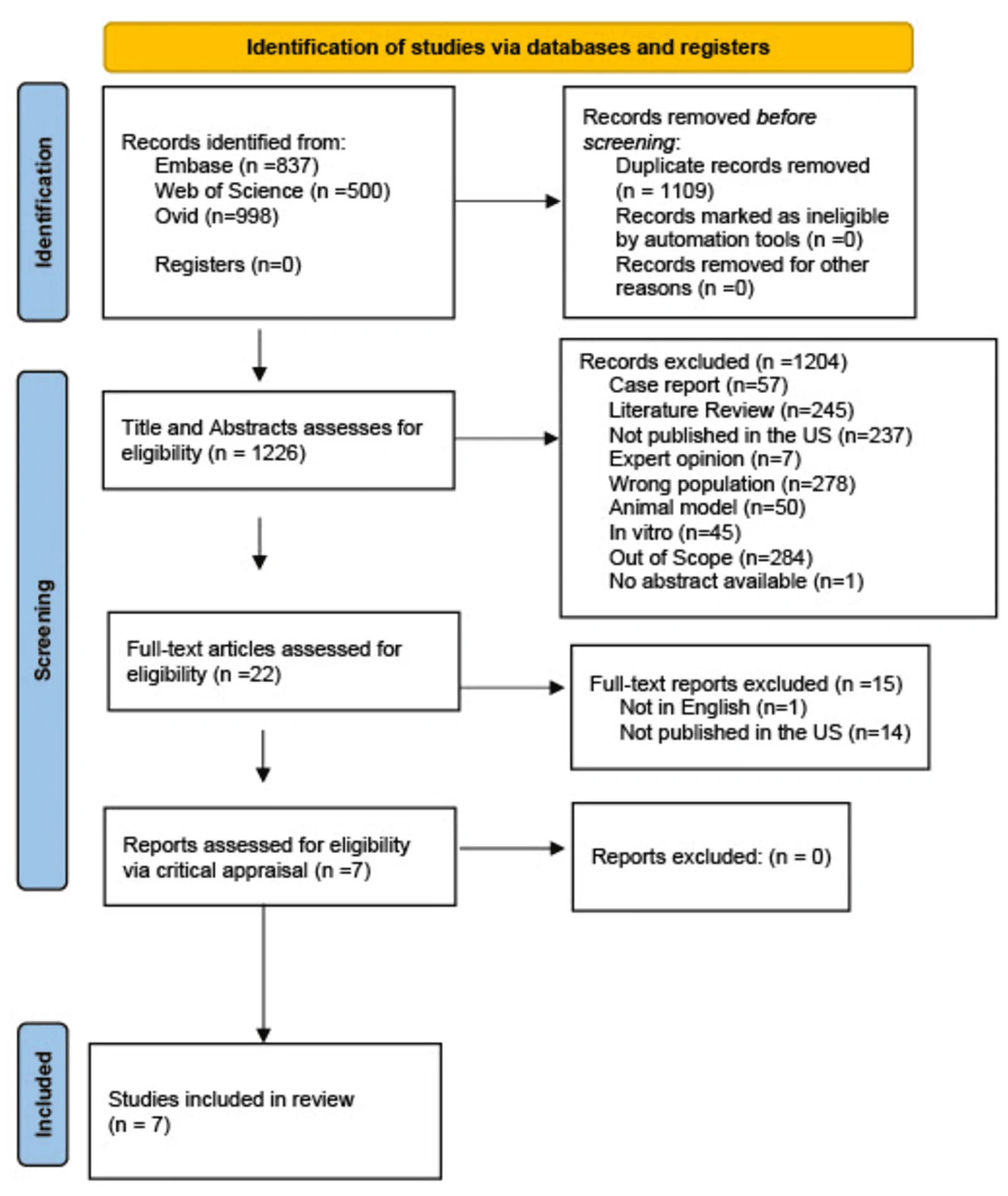
PRISMA-ScR PRISMA-ScR: Preferred Reporting Items for Systematic Reviews and Meta-Analyses extension for Scoping Reviews

Discussion

This scoping review aimed to assess the suitability of a booster dose of the measles vaccine for immunocompromised adults. From the studies surveyed, three main themes emerged: younger individuals had a higher rate of seronegativity, limitations in the completeness and accuracy of electronic health records (EHRs), and a lack of public health guidelines on measles vaccination and booster doses among adults [23,24].

Three of the studies identified higher rates of measles seronegativity among those born after 1957, a trend that the Centers for Disease Control and Prevention (CDC) attributes to reduced measles exposure and, thus, reduced natural measles immunity since the introduction of the monovalent measles vaccine in 1963 [24].

Lack of written documentation in the EHR was another key issue, as vaccination history was self-reported by patients, and patient recall did not determine measles immunity status. In most patients, measles vaccination history could not be determined [25].

Public health guidelines on measles vaccination provided limited guidance on measles vaccination schedules and seronegativity criteria, often lacking specifics for allogeneic hematopoietic cell transplant recipients or HIV patients. Two of the studies assessed live vaccine administration in immunocompromised patients, a practice normally contraindicated due to the risk of infection, and both found a low rate of adverse reactions and no association with breakthrough infection. These studies support the need for a study to determine the suitability of a measles vaccine booster dose in an adult immunocompromised population, as their increased rates of seronegativity and immunocompromised status put them at risk for an undue measles disease burden. This could be done by analyzing immune memory cells before and after booster administration [24,25].

The findings from this review demonstrated that only 70% of HIV-positive individuals were seropositive for measles immunity. This seropositivity rate is below the approximately 95% population immunity threshold associated with measles herd protection [23]. The similarity of these findings is likely due to a combination of natural waning of vaccine efficacy over time and weakened immune responses in immunocompromised individuals. Additionally, prior literature indicates that MMRV in immunocompromised adults has not been associated with severe adverse effects [28,29]. Since humoral and cell-mediated immunity were compromised in our review population, further studies could determine whether other immunotherapy approaches, such as a booster vaccine, could help prevent measles among vulnerable individuals [27].

Our initial findings warrant further research, but our review process was limited in several ways. Our research effort only included three databases, English-language publications, and studies conducted in the U.S. As such, studies that may have offered pertinent information were excluded if they were conducted in other countries, were not published in English, or were available in a database not used. There was limited generalizability because each study was conducted at a single clinic and involved a small sample of patients. Additionally, 57 of the 1,226 studies analyzed were observational, making it difficult to establish causation. Furthermore, some studies had incomplete patient immunization histories, which limited interpretation and may have introduced bias; in studies involving self-reported MMRV, recall bias may have been introduced. One study administered the MMRV vaccine to eligible patients based on treating physicians’ judgment, potentially biasing the findings by including healthier patients [26].

Several gaps in our studied populations were identified, particularly in underrepresented populations. Six of seven studies focused on immunocompromised populations with HIV, cell transplants, and IBD. Future research should focus on assessing the implications of boosting diverse immunocompromised populations with the MMRV. Based on the included populations, it is unknown whether older immunocompromised individuals who were seronegative would benefit from an MMRV booster, at the prescribing physician's discretion, to reduce disease burden. More studies are needed on additional measles immunotherapies for previously immunocompromised individuals [23,29].

## Conclusions

The results of this scoping review emphasized the need for future studies to support clear guidelines and policies regarding measles serologic screening and the administration of MMRV booster doses among immunocompromised individuals. This scoping review assessed the existing literature on measles immunity among individuals with immunocompromising conditions following vaccination. Specifically, there is an increasing need for further research to provide evidence to inform guidelines on the safety and use of booster vaccination in this population. Future studies should aim to include larger sample sizes and more diverse patient populations, encompassing a variety of immunocompromising conditions. Additionally, future studies should investigate the optimal timing of measles vaccine administration. This also underscores the importance of understanding the vaccine's safety profile across different immunocompromised states.

## Appendices

Appendix A

**Table 2:** Embase (N = 837)

No.	Query	Results
#1	measles:ti,ab,kw OR rubeola:ti,ab,kw OR 'measles mumps rubella':ti,ab,kw OR 'measles-mumps-rubella':ti,ab,kw OR mmr:ti,ab,kw OR mmrv:ti,ab,kw OR 'measles-containing':ti,ab,kw	837
#2	vaccin*:ti,ab,kw OR immuni*:ti,ab,kw	1866
#3	booster*:ti,ab,kw OR 'booster dose*':ti,ab,kw OR 'additional dose*':ti,ab,kw OR 'supplemental dose*':ti,ab,kw OR 'supplementary dose*':ti,ab,kw OR 'repeat dose*':ti,ab,kw OR revaccinat*:ti,ab,kw OR reimmuni*:ti,ab,kw OR 're immuni*':ti,ab,kw OR 'second dose':ti,ab,kw OR 'third dose':ti,ab,kw	3843
#4	(('bone marrow' NEAR/3 transplant*):ti,ab,kw) OR (('solid organ' NEAR/2 transplant*):ti,ab,kw) OR sot:ti,ab,kw OR hiv:ti,ab,kw OR aids:ti,ab,kw OR 'human immunodeficiency virus':ti,ab,kw OR cd4:ti,ab,kw OR autoimmune:ti,ab,kw OR 'auto-immune':ti,ab,kw OR 'inflammatory bowel':ti,ab,kw OR ibd:ti,ab,kw OR crohn*:ti,ab,kw OR 'ulcerative colitis':ti,ab,kw OR rheumatoid:ti,ab,kw OR lupus:ti,ab,kw OR sle:ti,ab,kw OR psoriasis:ti,ab,kw OR 'psoriatic arthritis':ti,ab,kw OR 'multiple sclerosis':ti,ab,kw OR vasculitis:ti,ab,kw OR biologic*:ti,ab,kw OR 'anti- tnf':ti,ab,kw OR adalimumab:ti,ab,kw OR infliximab:ti,ab,kw OR certolizumab:ti,ab,kw OR golimumab:ti,ab,kw OR etanercept:ti,ab,kw OR 'anti-cd20':ti,ab,kw OR rituximab:ti,ab,kw OR ocrelizumab:ti,ab,kw OR ofatumumab:ti,ab,kw OR belimumab:ti,ab,kw OR vedolizumab:ti,ab,kw OR ustekinumab:ti,ab,kw OR tocilizumab:ti,ab,kw OR abatacept:ti,ab,kw OR secukinumab:ti,ab,kw OR ixekizumab:ti,ab,kw OR guselkumab:ti,ab,kw OR tildrakizumab:ti,ab,kw OR 'jak inhibitor*':ti,ab,kw OR tofacitinib:ti,ab,kw OR baricitinib:ti,ab,kw OR upadacitinib:ti,ab,kw OR steroid*:ti,ab,kw OR corticosteroid*:ti,ab,kw OR prednisone:ti,ab,kw OR prednisolone:ti,ab,kw OR dexamethasone:ti,ab,kw OR cirrhosis:ti,ab,kw OR 'chronic kidney':ti,ab,kw OR ckd:ti,ab,kw OR esrd:ti,ab,kw OR dialysis:ti,ab,kw OR diabetes:ti,ab,kw OR 'end-stage renal':ti,ab,kw OR 'renal failure':ti,ab,kw	1017259
#5	adult*:ti,ab,kw OR 'aged':ti,ab,kw OR elder*:ti,ab,kw OR 'young adult*':ti,ab,kw OR 'middle age*':ti,ab,kw OR 'middle-aged':ti,ab,kw	3807088
#6	#2 OR #3	10281001
#7	#1 AND #4 AND #6	60908
#8	#7 AND ('article'/it OR 'article in press'/it)	991191
#9	#7 AND ('article'/it OR 'article in press'/it) AND 2015-2025/py	52628

Appendix B

**Table 3:** Ovid MEDLINE(R) (N = 998)

1	(measles or rubeola or "measles mumps rubella" or "measles-mumps- rubella" or MMR or MMRV or "measles-containing").ti,ab,kw.	37665
2	(vaccin* or immuni*).ti,ab,kw.	772550
3	(booster* or "booster dose*" or "additional dose*" or "supplemental dose*" or "supplementary dose*" or "repeat dose*" or revaccinat* or reimmuni* or "re immuni*" or "second dose" or "third dose").ti,ab,kw.	39491
4	(immunocompromis* or immuno-compromis* or immunodeficien* or immuno-deficien* or immunosuppress* or "immune suppress*" or cancer or oncolog* or chemotherap* or radiotherap* or transplant* or HSCT or ("stem cell" adj3 transplant*) or ("bone marrow" adj3 transplant*) or ("solid organ" adj2 transplant*) or SOT or HIV or AIDS or "human immunodeficiency virus" or CD4 or autoimmune or "auto-immune" or "inflammatory bowel" or IBD or Crohn* or "ulcerative colitis" or rheumatoid or lupus or SLE or psoriasis or "psoriatic arthritis" or "multiple sclerosis" or vasculitis or biologic* or "anti-TNF" or adalimumab or infliximab or certolizumab or golimumab or etanercept or "anti-CD20" or rituximab or ocrelizumab or ofatumumab or belimumab or vedolizumab or ustekinumab or tocilizumab or abatacept or secukinumab or ixekizumab or guselkumab or tildrakizumab or "JAK inhibitor*" or tofacitinib or baricitinib or upadacitinib or steroid* or corticosteroid* or prednisone or prednisolone or dexamethasone or cirrhosis or "chronic kidney" or CKD or ESRD or dialysis or diabetes or "end-stage renal" or "renal failure").ti,ab,kw.	7025378
5	2 or 3	786686
6	1 and 4 and 5	2454
7	limit 6 to (english language and yr="2015 - 2025" and journal article)	998

Appendix C

**Table 4:** Web of Science Core Collection (N = 1,085)

#	Search query	Results
1	TS=( measles OR rubeola OR "measles mumps rubella" OR "measles- mumps-rubella" OR MMR OR MMRV OR "measles-containing" )	39892
2	TS=((vaccin* OR immuni*) OR (booster* OR "booster dose*" OR "additional dose*" OR "supplemental dose*" OR "supplementary dose*" OR "repeat dose*" OR revaccinat* OR reimmuni* OR "re immuni*" OR "second dose" OR "third dose"))	951790
3	TS=( immunocompromis* OR immunodeficien* OR immunosuppress* OR "immune suppress*" OR cancer OR oncolog* OR chemotherap* OR radiotherap* OR transplant* OR HSCT OR ("stem cell" NEAR/3 transplant*) OR ("bone marrow" NEAR/3 transplant*) OR ("solid organ" NEAR/2 transplant*) OR SOT OR HIV OR AIDS OR "human immunodeficiency virus" OR CD4 OR autoimmune OR "auto-immune" OR "inflammatory bowel" OR IBD OR Crohn* OR "ulcerative colitis" OR rheumatoid OR lupus OR SLE OR psoriasis OR "psoriatic arthritis" OR "multiple sclerosis" OR vasculitis OR biologic* OR "anti-TNF" OR adalimumab OR infliximab OR certolizumab OR golimumab OR etanercept OR "anti-CD20" OR rituximab OR ocrelizumab OR ofatumumab OR belimumab OR vedolizumab OR ustekinumab OR tocilizumab OR abatacept OR secukinumab OR ixekizumab OR guselkumab OR tildrakizumab OR "JAK inhibitor*" OR tofacitinib OR baricitinib OR upadacitinib OR steroid* OR corticosteroid* OR prednisone OR prednisolone OR dexamethasone OR cirrhosis OR "chronic kidney" OR CKD OR ESRD OR dialysis OR diabetes OR "end-stage renal" OR "renal failure" )	9939361
4	#3 AND #2 AND #1	3485
5	#3 AND #2 AND #1 and Article (Document Types)	2524
6	#3 AND #2 AND #1 and Article (Document Types) and English (Languages)	2434
7	#3 AND #2 AND #1 and Article (Document Types) and English (Languages) and 2025 or 2024 or 2023 or 2022 or 2021 or 2020 or 2018 or 2017 or 2019 or 2016 or 2015 (Publication Years)	1085
